# Zebrafish (Danio rerio) as a Vertebrate Model Host To Study Colonization, Pathogenesis, and Transmission of Foodborne Escherichia coli O157

**DOI:** 10.1128/mSphereDirect.00365-17

**Published:** 2017-09-20

**Authors:** Daniel H. Stones, Alexander G. J. Fehr, Laurel Thompson, Jacqueline Rocha, Nicolas Perez-Soto, Vipin T. P. Madhavan, Kerstin Voelz, Anne Marie Krachler

**Affiliations:** a University of Birmingham, School of Biosciences, Institute of Microbiology and Infection, Edgbaston, Birmingham, United Kingdom; b Department of Microbiology and Molecular Genetics, University of Texas McGovern Medical School at Houston, Houston, Texas, USA; c MD Anderson and UTHealth Graduate School of Biomedical Science, Houston, Texas, USA; University of Kentucky; Imperial College London; Leiden University

**Keywords:** EHEC, O157, foodborne pathogens, gastrointestinal infection, infection model, intravital imaging, zebrafish

## Abstract

Foodborne infections with enterohemorrhagic Escherichia coli (EHEC) are a major cause of diarrheal illness in humans and can lead to severe complications such as hemolytic uremic syndrome. Cattle and other ruminants are the main reservoir of EHEC, which enters the food chain through contaminated meat, dairy, or vegetables. Here, we describe the establishment of a vertebrate model for foodborne EHEC infection, using larval zebrafish (Danio rerio) as a host and the protozoan prey *Paramecium caudatum* as a vehicle. We follow pathogen release from the vehicle, intestinal colonization, microbe-host interactions, and microbial gene induction within a live vertebrate host, in real time, throughout the course of infection. We demonstrate that foodborne EHEC colonizes the gastrointestinal tract faster and establishes a higher burden than waterborne infection. Expression of the locus of enterocyte effacement (LEE), a key EHEC virulence factor, was observed early during infection, mainly at sites that experience fluid shear, and required tight control to enable successful host colonization. EHEC infection led to strain- and LEE-dependent mortality in the zebrafish host. Despite the presence of the endogenous microbiota limiting EHEC colonization levels, EHEC colonization and virulence can be studied either under gnotobiotic conditions or against the backdrop of an endogenous (and variable) host microbiota. Finally, we show that the model can be used for investigation of factors affecting shedding and transmission of bacteria to naive hosts. Overall, this constitutes a useful model, which ideally complements the strengths of existing EHEC vertebrate models.

**IMPORTANCE** Enterohemorrhagic Escherichia coli (EHEC) is a foodborne pathogen which can cause diarrhea, vomiting, and, in some cases, severe complications such as kidney failure in humans. Up to 30% of cattle are colonized with EHEC, which can enter the food chain through contaminated meat, dairy, and vegetables. In order to control infections and stop transmission, it is important to understand what factors allow EHEC to colonize its hosts, cause virulence, and aid transmission. Since this cannot be systematically studied in humans, it is important to develop animal models of infection and transmission. We developed a model which allows us to study foodborne infection in zebrafish, a vertebrate host that is transparent and genetically tractable. Our results show that foodborne infection is more efficient than waterborne infection and that the locus of enterocyte effacement is a key virulence determinant in the zebrafish model. It is induced early during infection, and loss of tight LEE regulation leads to a decreased bacterial burden and decreased host mortality. Overall, the zebrafish model allows us to study foodborne infection, including pathogen release from the food vehicle and gene regulation and its context of host-microbe interactions, as well as environmental shedding and transmission to naive hosts.

## Abstract (precis)

Enterohemorrhagic Escherichia coli (EHEC) is a foodborne pathogen which can cause diarrhea, vomiting, and, in some cases, severe complications such as kidney failure in humans. Up to 30% of cattle are colonized with EHEC, which can enter the food chain through contaminated meat, dairy, and vegetables. In order to control infections and stop transmission, it is important to understand what factors allow EHEC to colonize its hosts, cause virulence, and aid transmission. Since this cannot be systematically studied in humans, it is important to develop animal models of infection and transmission. We developed a model which allows us to study foodborne infection in zebrafish, a vertebrate host that is transparent and genetically tractable. Our results show that foodborne infection is more efficient than waterborne infection and that the locus of enterocyte effacement is a key virulence determinant in the zebrafish model. It is induced early during infection, and loss of tight LEE regulation leads to a decreased bacterial burden and decreased host mortality. Overall, the zebrafish model allows us to study foodborne infection, including pathogen release from the food vehicle and gene regulation and its context of host-microbe interactions, as well as environmental shedding and transmission to naive hosts.

## INTRODUCTION

Enterohemorrhagic Escherichia coli (EHEC) is a major cause of foodborne infections worldwide. EHEC is transmitted through consumption of water, meat, dairy, or vegetables contaminated with fecal matter or is transmitted hand to mouth, which is common in school and nursery settings. EHEC infection usually presents with bloody diarrhea, vomiting, and stomach cramps but in rare cases can lead to hemolytic uremic syndrome (HUS), a severe clinical complication resulting in kidney damage and often in lifelong morbidity or in mortality. Antibiotics are contraindicated, since antibiotic treatment can increase toxin production and exacerbate toxin-mediated disease pathology. Depending on geographical location, up to 30% of cattle are colonized by EHEC, which represents a considerable environmental reservoir. One of the main virulence factors associated with colonization of ruminants as well as human hosts is the locus of enterocyte effacement (LEE), a horizontally acquired pathogenicity island encoding a type 3 secretion system (T3SS). The LEE also encodes the adhesion intimin and its host translocated receptor Tir (translocated intimin receptor), which in volunteer studies performed with the closely related enteropathogenic E. coli (EPEC) has been shown to be a key factor for the development of diarrheal symptoms (1).

Ongoing studies of EHEC focus on understanding how the LEE is regulated during the EHEC life cycle and how the LEE-carried genes and other virulence factors, such as Shiga toxin (Stx), contribute to colonization, disease pathogenesis, and transmission. Another area of interest is how a host’s endogenous microbiota interacts with EHEC and how this affects host fate following EHEC ingestion. Several infection models exist to study EHEC virulence factors, most notably pigs, rabbits, and mice. Although none of these model hosts is capable of reproducing the full clinical presentation of human EHEC infection, each has its own distinct advantages and disadvantages (2). Gnotobiotic piglets inoculated with EHEC present a model for gastrointestinal (GI) tract pathology but fail to develop systemic symptoms. Additionally, this model is expensive, requires dedicated and highly specialized facilities, and is not particularly genetically tractable. Inoculation of neonate or infant rabbits through gastric catheters leads to colonization, diarrhea, and GI tract histopathology that resembles that of human infection but does not lead to mortality (3–5). Mice present the least expensive and most widely available vertebrate infection model to date, but their endogenous microbiota prevents EHEC colonization and has to be removed by streptomycin treatment to allow infection. Streptomycin-treated mice can be used to study colonization but fail to develop diarrhea, colitis, or attaching and effacing (A/E) lesions (6). All three models present limited opportunities to study phenotypical changes in microbe and host simultaneously, in real time, and over a prolonged period. Intravital imaging of intestinal infection in these vertebrate models is possible but challenging, since part of the intestine has to be surgically exposed, which constitutes a major procedure and can be done for only a limited duration. Additionally, it is technically challenging to study host-microbe interactions at the single-cell level in this context.

Here, we set out to study EHEC colonization and host-microbe interactions and transmission in zebrafish (Danio rerio), a vertebrate host that is inexpensive, gives rise to a large number of offspring with short generation times, and is genetically tractable and transparent during infancy. Due to these features, zebrafish larvae have become a well-established model for many bacterial (7–9), viral (10), and fungal (11) infections and for pathogen-microbiota interactions (12, 13). Paired with the transparency of zebrafish, its genetic tractability means that phenotypical features of both microbe and host, and their changes in response to interactions, can be studied during infection within a live host, over extended periods, and in a high-throughput format (14, 15). Changes in microbial and host gene expression, in immune cell recruitment, and in host tissue morphology and intracellular signaling thus all become directly observable (16).

We capitalized on these features to study the dynamics of colonization and pathogen-microbiota interactions and the effect of previously described virulence factors on microbe-host interactions following foodborne infection of infant zebrafish with EHEC. We demonstrate that the route of infection significantly affects the level of EHEC colonization, that EHEC virulence factors are active early in the infection process, and that they require precise control in order for EHEC to colonize effectively. In addition, we demonstrate the ability of EHEC to significantly increase neutrophil migration to the gut and surrounding tissue compared to the levels seen with uninfected fish. Finally, we show that the zebrafish model can be used to study the role of endogenous microbiota during infection and the dynamics of fecal transmission.

## RESULTS

### *Paramecium caudatum* acts as a vehicle for Escherichia coli.

**FIG 1:**
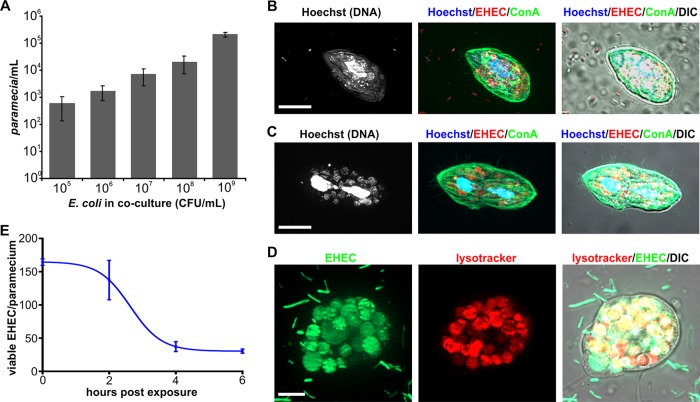
*Paramecium caudatum* acts as a vector for Escherichia coli. (A) EHEC and *P. caudatum* were grown in coculture for 16 h, and *P. caudatum* numbers were measured using a hemocytometer. Numbers of paramecia at the end of the experiment were graphed against initial bacterial concentrations (in CFU per milliliter). Values shown are means ± standard deviations (SD) (*n* = 3). (B) Following 2 h of coincubation with EHEC::mCherry (red), *P. caudatum* samples were fixed and subjected to Hoechst staining (DNA, blue) and concanavalin A (ConA) staining (green). (C) Some paramecia were proliferating on a diet of EHEC and were undergoing cell division. Scale bars, 10 μm. (D) Following 2 h of coculture with EHEC::gfp (green), *P. caudatum* was incubated with lysotracker (red) to visualize vacuole acidification. Scale bar, 5 μm. (E) Following 2 h of coincubation with EHEC, *P. caudatum* was transferred to medium without bacteria. Samples were removed at the indicated time points, and numbers of viable E. coli cells within paramecia were determined by dilution plating on selective agar (*n* = 3).

Existing animal models predominantly administer E. coli as liquid suspension, through orogavage (2). In contrast, infections in humans usually result from ingestion of contaminated food. The unicellular ciliated protozoan *Paramecium caudatum* is used as a natural food source that zebrafish larvae prey upon. It is abundant in freshwater and in brackish and marine water and has been described as a reservoir for environmental bacteria (17) as well as a vector for fish diseases (18). Thus, we tested whether it could be established as a vector for foodborne infection of zebrafish larvae with EHEC. *P. caudatum* preys on bacteria, algae, or yeasts, which it takes up via its ciliated oral groove and delivers into its mouth opening, from which the ingested material reaches the gullet. Eventually, the ingested material blebs and is internalized into a vacuole, which gradually acidifies and renders the contents subject to degradation. We studied the interactions between *P. caudatum* and the enterohemorrhagic E. coli O157:H7 Sakai strain to establish if and for how long bacteria would persist within *Paramecium*. *P. caudatum* was able to utilize E. coli as a food source, as paramecia kept in coculture proliferated in a dose-dependent manner (Fig. 1A). Fluorescence imaging following coculture with EHEC::mCherry revealed that the paramecia contained E. coli, which was localized within food vacuoles (Fig. 1B to D). Ingestion of EHEC did not seem to have any adverse effects on *P. caudatum* (Fig. 1B), and, indeed, paramecia were observed to proliferate on a diet of EHEC (Fig. 1C). Further investigation using coculture of *P. caudatum* and strain EHEC::*gfp* revealed that E. coli-containing food vacuoles remained transiently stable but eventually acidified (Fig. 1D), leading to degradation of E. coli. The time course of degradation was determined by plating of viable E. coli recovered from paramecium (Fig. 1E). Ingested E. coli was degraded with a half-life of approximately 2.5 h (Fig. 1E). This aligns with our visual confirmation of EHEC degradation within paramecia (see Fig. S1 in the supplemental material). Dilution plating of EHEC-containing paramecia grown on EHEC for 2 h showed that each *P. caudatum* contained an average of 200 viable E. coli cells and that the number of intracellular EHEC was independent of bacterial density over a concentration range of 2.5 × 10^7^ to 1 × 10^8^ CFU/ml. This allowed us to establish *P. caudatum* as a vector to administer a defined dose of foodborne EHEC to zebrafish larvae.

10.1128/mSphereDirect.00365-17.1 FIG S1 Degradation of internalized EHEC by *P. caudatum*. At specified time points, paramecia incubated with EHEC Sakai::mCherry were washed, fixed in 4% paraformaldehyde (PFA), mounted in antifade gold, and imaged using a Zeiss Axio Observer inverse microscope. Download FIG S1, TIF file, 0.4 MB. Copyright © 2017 Stones et al. 2017 Stones et al. This content is distributed under the terms of the Creative Commons Attribution 4.0 International license.

### Foodborne E. coli colonizes larval zebrafish more efficiently than waterborne EHEC.

**FIG 2:**
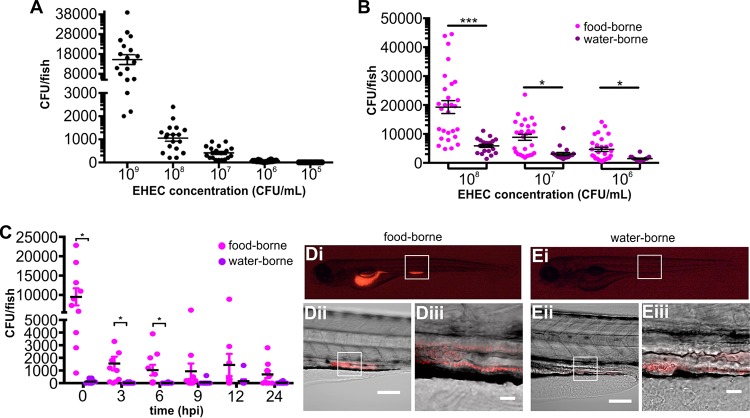
Colonization of larval zebrafish by waterborne and foodborne EHEC. (A) Waterborne EHEC infection. Zebrafish larvae were exposed to EHEC for 16 h at the indicated concentrations. Following exposure, fish were washed thoroughly and homogenized and CFU counts were determined by dilution plating on EHEC selective agar. Individual data points (*n* = 18 for each concentration) and means and SD are shown. (B) Zebrafish larvae were exposed to foodborne (magenta) or waterborne (purple) EHEC for 2 h, at the indicated doses. The bacterial burden was determined by dilution plating on EHEC selective agar. Individual data points (*n* = 28 for each condition), means, and SD are shown. (C) Zebrafish larvae were exposed to 10^8^ CFU/ml of foodborne (magenta) or waterborne (purple) EHEC for 2 h and transferred to fresh sterile medium, and bacterial burden was determined by dilution plating on EHEC selective agar at the indicated time points. Individual data points, means (*n* = 10 for each condition), and SD are shown. Statistical significance was calculated using the Mann-Whitney *U* test (***, *P* < 0.005; **; *P* < 0.01; *, *P* < 0.05). (D and E) Live images of zebrafish larvae exposed to foodborne (D) or waterborne (E) EHEC::mCherry (10^8^ CFU/ml) for 2 h. A whole embryo (i) and a magnified section of a mid-intestinal region are shown at magnifications of ×20 (ii) and ×64 (iii). Scale bars in panels D and E are 100 μm and 20 μm for panels ii and iii, respectively.

Initially, colonization by waterborne EHEC was characterized in gnotobiotic zebrafish larvae, which were acquired from bleached eggs and reared under sterile conditions as previously described (19). EHEC was administered to larval zebrafish at 4 days postfertilization (dpf), at which point larvae had a fully developed intestinal tract with a functional anal opening. E. coli constitutively expressing mCherry was used to visualize colonization *in vivo*. Bacterial burden was initially assessed following 16 h of exposure and increased in a manner dependent on the amount of ingested EHEC (Fig. 2A).

Next, paramecia loaded with EHEC Sakai::mCherry were administered to larval zebrafish at 4 dpf, at which point larvae were able to swim freely and to prey on paramecium (see Fig. S2 and Movie S1 in the supplemental material). To administer a consistent number of E. coli cells to zebrafish, paramecia were kept in coculture with E. coli at a ratio of 1:10^4^, gently washed to remove extracellular E. coli, added to fish medium to result in a defined initial concentration as described above, and offered to zebrafish as a prey. Monitoring of paramecia prestained with the lipophilic dye BacLight green and then fed with EHEC::mCherry within live zebrafish demonstrated that paramecia reached the intestinal bulb intact and were then degraded and released EHEC::mCherry bacteria (Fig. S2B).

10.1128/mSphereDirect.00365-17.2 FIG S2 (A) Stills from Movie S1, showing a zebrafish larva at 4 dpf preying on *P. caudatum* loaded with EHEC Sakai::mCherry. (B) Baclight green-stained paramecia (green) containing EHEC::mCherry (red) inside the foregut of a live zebrafish at 2 h postinfection, following mounting in 1% low-melting-temperature agarose and imaging on an Olympus IX83 microscope with a Fluoview FW3000 confocal system. Download FIG S2, TIF file, 0.7 MB. Copyright © 2017 Stones et al. 2017 Stones et al. This content is distributed under the terms of the Creative Commons Attribution 4.0 International license.

10.1128/mSphereDirect.00365-17.3 MOVIE S1 A zebrafish larva at 4 dpf preying on *P. caudatum* loaded with EHEC Sakai::mCherry. Download MOVIE S1, MOV file, 14 MB. Copyright © 2017 Stones et al. 2017 Stones et al. This content is distributed under the terms of the Creative Commons Attribution 4.0 International license.

To assess the differences in the levels of colonization by the foodborne and waterborne infection routes, bacterial burden was determined in fish exposed to matched doses of foodborne or waterborne EHEC following 2 h of exposure. At all three doses tested, colonization levels were approximately 10 times higher following foodborne infection (Fig. 2B). Foodborne infection also led to increased persistence of EHEC in the intestinal tract; while waterborne EHEC was gradually cleared by most fish over the course of 24 h, foodborne EHEC persisted at high levels up to 24 h postinfection (hpi) in approximately 50% of the fish, while the other half carried lower burdens or cleared the infection over the same time span (Fig. 2C).

**FIG 3:**
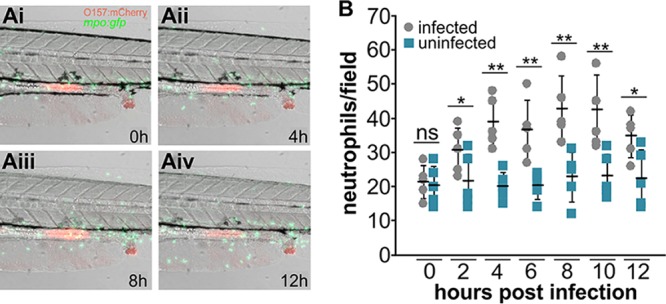
EHEC triggers transient neutrophil recruitment to the intestinal region and surrounding tissue upon colonization. *mpo*::*gfp* zebrafish larvae were exposed to 10^8^ CFU/ml foodborne EHEC::mCherry for 2 h, washed, mounted live in low-melting-temperature agarose-containing tricaine, and imaged for 12 h at 32°C. (A) Representative frames showing intestinal tract of infected fish after 0 h (Ai), 4 h (Aii), 8 h (Aiii), and 12 h (Aiv). Accumulation of fecal matter at the anal opening is also visible. (B) Neutrophils in the intestinal region and surrounding tissue (counts from the intestinal region as shown in panel A) were enumerated. Individual data points from infected fish (gray) and uninfected fish (blue), means (*n* = 5 per condition), and SD are shown. The statistical significance of results of comparisons between infected and uninfected fish for individual time points was determined using Student’s *t* test (*, *P* < 0.05; **, *P* < 0.01). ns, not significant.

Analysis of EHEC colonization revealed a strong presence of EHEC in the intestinal bulb of the larvae subjected to foodborne infection (Fig. 2Di), although this was transient, with more-persistent colonization mainly localized to the mid-intestine (Fig. 2D and E). Accumulation and secretion of EHEC-containing fecal matter were also observed soon after ingestion (Fig. 3A).

### EHEC triggers transient neutrophil recruitment to the intestinal region and surrounding tissue upon colonization.

EHEC infection causes release of interleukin-8 (IL-8), a potent neutrophil chemoattractant, by the intestinal mucosa (20). Humans infected with EHEC shed a high number of leukocytes in their feces (21), and tissue biopsy specimens taken from patients infected with EHEC revealed a severe inflammatory response to EHEC and accompanying damage of the tissue in the cecum and colon (22, 23). To test whether EHEC colonization triggers a proinflammatory response in zebrafish, we followed neutrophil movement in infected transgenic larvae [Tg(*mpo*::*egfp*)^i114^] featuring green fluorescent protein (GFP)-expressing neutrophils (24). Rapid neutrophil recruitment to the intestinal region and surrounding tissue was observed in infected animals but not in uninfected control animals (Fig. 3). Neutrophils were especially concentrated in areas adjacent to the mid-intestinal infection focus and anal region (Fig. 3Aiii). Recruitment was significantly elevated compared to uninfected control results at 2 hpi and peaked around 8 hpi (Fig. 3B).

### EHEC induces the locus of enterocyte effacement at the site of colonization.

The locus of enterocyte effacement (LEE) is a horizontally acquired pathogenicity island encoding structural and regulatory components of a type 3 secretion system (T3SS). It is a feature shared by A/E lesion-producing pathogens, including enteropathogenic E. coli (EPEC), Citrobacter rodentium, and EHEC, and contributes to intestinal persistence and disease severity in animal models (5, 25). Expression of LEE-carried genes is silenced by H-NS (histone-like nucleoid structuring protein) under environmental conditions (26) and induced under physiological conditions mimicking the host intestinal tract (27). The LEE is organized in five transcriptional units, which require the LEE1-encoded master regulator Ler (LEE-encoded regulator) for induction (28). Using an EHEC::mCherry reporter strain carrying the LEE1 promoter driving GFP expression, we investigated if and where this virulence factor is expressed by EHEC following ingestion by zebrafish.

**FIG 4:**
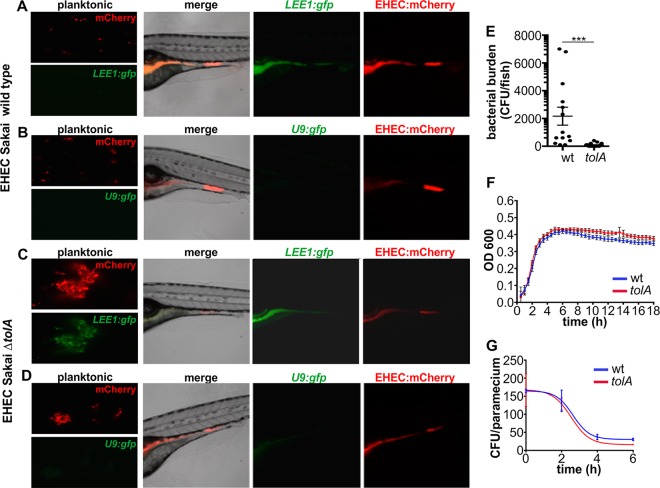
EHEC induced the locus of enterocyte effacement at the site of colonization. (A) Micrograph of Sakai wild-type (wt) *EHEC*::*mCherry* cotransformed with *LEE1*::*gfp* grown planktonically in E3 medium and zebrafish colonization by *EHEC*::*mCherry*/*LEE1*::*gfp*. Also see Movie S2 for a time-lapse presentation of the course of the infection shown in panel A. (B) Wild-type EHEC::mCherry cotransformed with promoterless control U9::*gfp* grown planktonically and zebrafish colonization by EHEC::mCherry cotransformed with U9::*gfp*. Zebrafish were exposed to 10^8^ CFU/ml of food-borne EHEC for 2 h. (C) EHEC Sakai isogenic *ΔtolA* mutant transformed with mCherry and *LEE1*::*gfp* grown planktonically and during zebrafish colonization. (D) EHEC Sakai isogenic Δ*tolA* mutant transformed with mCherry and promoterless *U9*::*gfp* grown planktonically and during zebrafish colonization. (E) Bacterial burden was determined by dilution plating on EHEC selective agar. Individual data points (*n* = 15 for each condition), means, and SD are shown. Statistical significance was determined using Student’s *t* test (***, *P* < 0.001). (F) Growth curves of the Sakai wild-type strain (blue) and the Δ*tolA* mutant (red). (G) Degradation profiles for the Sakai wild-type strain (blue) and the Δ*tolA* mutant (red) in *P. caudatum* as determined by lysis and dilution plating. Regression analysis showed that there is no difference between the slopes (*P* = 0.7016).

While EHEC LEE1::gfp showed low levels of fluorescence outside the host (Fig. 4A and Movie S2), GFP expression, and thus LEE induction, was observed in the zebrafish intestinal tract (Fig. 4A). In contrast, a control strain lacking a promoter driving GFP expression formed similar infection foci but did not express GFP (Fig. 4B). This suggests that the LEE-encoded T3SS is induced early during colonization of the zebrafish gastrointestinal tract.

10.1128/mSphereDirect.00365-17.4 MOVIE S2 A zebrafish larva at 4 dpf infected with 10^8^ CFU/ml of foodborne Sakai wild-type EHEC::mCherry cotransformed with *LEE1*::*gfp*. The movie shows the progression of colonization and LEE1 induction over 20 hpi. Download MOVIE S2, AVI file, 3.6 MB. Copyright © 2017 Stones et al. 2017 Stones et al. This content is distributed under the terms of the Creative Commons Attribution 4.0 International license.

### Fine-tuning of LEE expression is required for successful colonization.

Since we observed LEE expression in the zebrafish intestinal tract, we next tested if LEE expression and EHEC intestinal colonization levels were linked, using the zebrafish model. We first set out to test the effect of enhanced T3SS expression on colonization levels and persistence of EHEC in the zebrafish gut, using the *LEE1*::*gfp* reporter. The protein TolA is a component of the *trans*-envelope Tol system required for cell wall remodeling in E. coli (29). Loss of *tolA* in EHEC leads to activation of the Rcs phosphorelay and to constitutive activation of LEE-carried virulence genes (30). Despite a higher level of T3SS expression in the Δ*tolA* mutant, the adherence of the strain to intestinal epithelial cells and its virulence in the Galleria mellonella infection model are significantly reduced (30). We constructed a nonpolar *tolA* mutant in the Sakai background and compared its LEE induction levels and ability to colonize the zebrafish intestine to those of the wild-type Sakai strain following 2 h of infection. *LEE1*::*gfp* transcriptional activity was significantly higher in the *tolA* mutant grown planktonically in E3 medium (5 mM NaCl, 0.17 mM KCl, 0.33 mM CaCl_2_, 0.33 mM MgSO_4_) than in wild-type bacteria (Fig. 4C), as previously described (30). This also held true for the *tolA* mutant within the zebrafish intestinal tract (Fig. 4C). In accordance with previous models, the bacterial burden of the *tolA* mutant was significantly reduced compared to that of the wild type (Fig. 4E). However, this was not due to altered growth or degradation within the *P. caudatum* vector (Fig. 4F and G). Overall, these data suggest that the tight regulation of T3SS expression is essential for within-host fitness.

**FIG 5:**
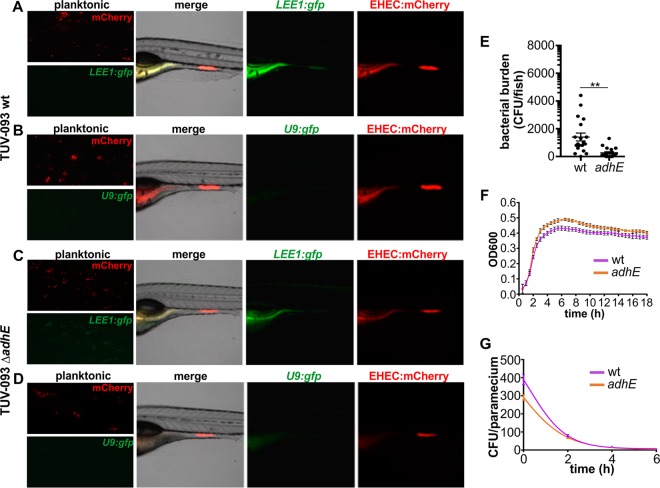
LEE expression and colonization of EHEC Δ*adhE*. (A) EHEC TUV 93-0 wild type transformed with mCherry and *LEE1*::*gfp* grown planktonically in E3 medium and in zebrafish. (B) EHEC TUV 93-0 wild type transformed with mCherry and promoterless* U9*::*gfp* grown planktonically and in zebrafish. (C) EHEC TUV 93-0 isogenic Δ*adhE* mutant transformed with mCherry and *LEE1*::*gfp* grown planktonically and in zebrafish. (D) EHEC TUV 93-0 isogenic Δ*adhE* mutant transformed with mCherry and promoterless* U9*::*gfp* grown planktonically and in zebrafish. (E) Bacterial burden was determined by dilution plating on EHEC selective agar. Individual data points (*n* = 15 for each condition), means, and SD are shown. Statistical significance was determined using Student’s *t* test (**, *P* < 0.01). (F) Growth curves of TUV 93-0 wild type (pink) and Δ*adhE* mutant (orange). Means (*n* = 3) and SD are shown. (G) Degradation profiles for TUV 93-0 wild type (pink) and Δ*adhE* mutant (orange) in *P. caudatum* as determined by lysis of *P. caudatum* and dilution plating. Regression analysis showed there is no difference between the slopes (*P* = 0.2909).

We next sought to investigate the effect of reduced T3SS expression on colonization and persistence of EHEC in the zebrafish gut. The metabolic enzyme AdhE, a bifunctional acetaldehyde-coenzyme A (acetaldehyde-CoA) dehydrogenase and alcohol dehydrogenase, regulates virulence gene expression in EHEC O157. Deletion of *adhE* leads to strong suppression of the T3SS (31). The *adhE* mutant was significantly less virulent than its parental strain, TUV 93-0, and showed approximately 10-fold-lower colonization levels in an infant rabbit infection model (31). These observations from the infant rabbit model were recapitulated in our zebrafish model; following a 2-h exposure to 10^8^ CFU/ml of foodborne EHEC, LEE1::gfp expression was significantly induced in the TUV 93-0 wild-type strain colonizing the intestinal tract compared to the results seen with planktonic growth (Fig. 5A and B). In contrast, reduced LEE1::gfp induction was observed in the* adhE* mutant both in planktonic culture and during host colonization (Fig. 5C and D). The bacterial burden was approximately 10-fold reduced for the *adhE* mutant compared to the TUV 93-0 wild type (Fig. 5E), although both growth (Fig. 5F) and persistence (Fig. 5G) within *P. caudatum* were unaltered.

### EHEC ingestion causes strain-specific mortality in zebrafish.

**FIG 6:**
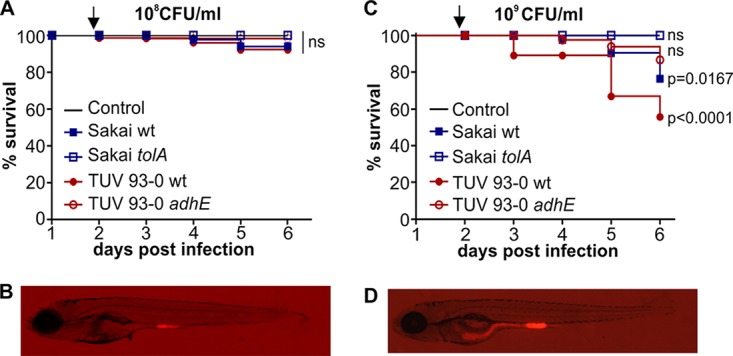
EHEC ingestion causes dose-dependent and strain-specific mortality in zebrafish. Zebrafish at 4 dpf were infected with 10^8^ CFU/ml (A and B) or 10^9^ CFU/ml (C and D) of EHEC (Sakai wt, blue full squares; Sakai *tolA*, empty squares; TUV 93-0 wt, red filled circles; TUV *adhE*, red empty circles) or left uninfected (black line below *tolA* data) and incubated at 32°C for up to 6 dpi. Fish were assessed for vital signs (presence of movement or heartbeat or circulation) every 24 h, and percent survival was plotted using Kaplan-Meier analysis (*n* = 15 fish per condition, over three independent experiments). Statistical significance was assessed using a Mantel-Cox test. Images of fish infected with EHEC wild-type Sakai::mCherry were taken at 21 hpi (as indicated by an arrow in each survival graph).

Having established that T3SS expression is specifically induced in the zebrafish host and is necessary for efficient intestinal colonization, we asked if EHEC ingestion would cause mortality in zebrafish. We infected zebrafish at 4 dpf with a dose of either 10^8^ CFU/ml or 10^9^ CFU/ml of waterborne EHEC or left them uninfected (control). Fish were assessed for vital signs (movement, heartbeat, and circulation) daily for a total of 6 days postinfection (dpi), and survival was analyzed using the Kaplan-Meier estimator (Fig. 6). At a challenge dose of 10^8^ CFU/ml, no significant mortality was observed. A dose of 10^9^ CFU/ml caused more-robust colonization and strain-specific mortality (Fig. 6C and D). Despite showing similar colonization levels (Fig. 4 and 5), the Sakai and TUV 93-0 wild-type strains displayed significantly different levels of pathogenicity, with mean survival rates of 76% for Sakai and 56% for TUV 93-0 at the experimental endpoint. The attenuation of the *tolA* and *adhE* mutants observed in terms of intestinal colonization (Fig. 4 and 5) was also reflected by a loss in pathogenicity. The *tolA* mutant was nonpathogenic in the zebrafish model, with 100% host survival and no observable morbidity up to day 6 postinfection. The *adhE* mutant was similarly attenuated, with a mean host survival rate of 87% at day 6 postinfection (Fig. 6).

### Zebrafish as a model to study fecal shedding and transmission.

**FIG 7:**
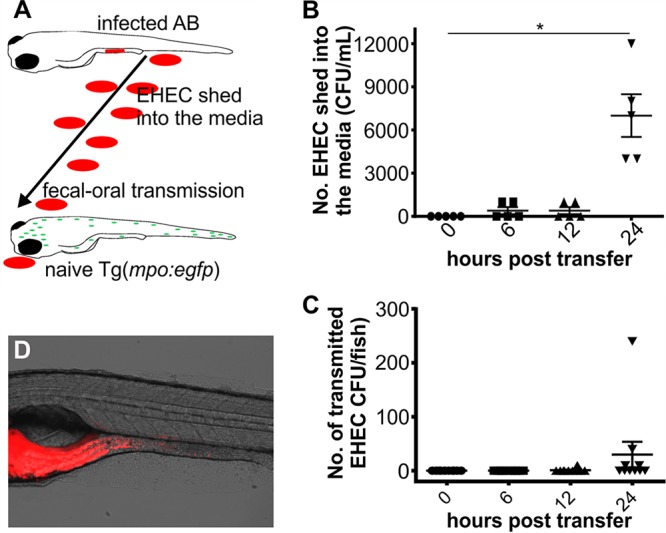
Zebrafish as a model to study fecal shedding and transmission. (A) Scheme depicting experimental setup of shedding and transmission experiment. Infected AB embryos were transferred into fresh media housing naive Tg(*mpo*::*egfp*) embryos (both 4 dpf). Posttransfer, shedding of EHEC into the media and transfer to recipient animals were monitored over time. (B) EHEC shed into the media were enumerated immediately and at 6, 12, and 24 h posttransfer of infected AB embryos into fresh media. Shown are individual data points, means, and SD (*n* = 5/time point). (C) The bacterial burden of Tg(*mpo*:*egfp*) recipient embryos was quantified immediately and at 6, 12, and 24 h posttransfer of infected AB (donor) embryos. Shown are individual data points, means, and SD (*n* = 10 embryos/time point). Statistical significance was calculated using the Kruskal-Wallis test with Dunn’s multiple-comparison test (*, *P* < 0.05). (D) Example of EHEC::mCherry localization in recipient embryos.

While fecal shedding of EHEC is often used as a proxy for bacterial burden in rodents, fecal-oral transmission is rarely studied in these models. The zebrafish model allows simultaneous analysis of fecal shedding and fecal-oral transmission from infected fish to naive recipients in one experiment. AB fish infected with foodborne EHEC for 2 h were transferred into fresh media together with a naive recipient (Fig. 7A). Tg(*mpo*:*gfp*) fish were used as recipients to be able to visually distinguish donors and recipients (green fluorescence). EHEC was continually shed from donor fish following transfer into fresh media, and levels of shed bacteria increased steadily up to 24 h posttransfer (Fig. 7B). Onward transmission to naive fish was first observed between 12 and 24 h posttransfer (Fig. 7C), at which point EHEC::mCherry could be visualized in the foregut and mid-intestine of recipient embryos (Fig. 7D). These data demonstrate that the dynamics of fecal shedding and fecal-oral transmission to naive hosts can be studied using the zebrafish model.

### The zebrafish microbiota provides a barrier to EHEC colonization.

To this point, our experiments were done in gnotobiotic zebrafish, as the endogenous microbiota often proves a significant barrier to colonization (32, 33). And yet the interactions between ingested pathogens and the endogenous microbiota, and the impact of the microbiota composition on the fate of infection, are of interest, particularly in the case of EHEC (34–36), and it would be desirable to be able to address these issues in the zebrafish infection model.

**FIG 8:**
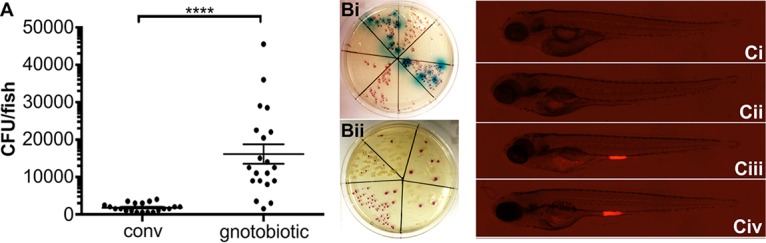
The zebrafish microbiota provides a barrier to E. coli colonization. (A) Following exposure of conventionalized (conv) or gnotobiotic zebrafish larvae to foodborne EHEC (10^8^ CFU/ml) for 2 h, bacterial burden was determined by dilution plating on EHEC selective agar. Individual data points, means (*n* = 20 for each condition), and SD are shown. Statistical significance was determined using Student’s *t* test (****, *P* < 0.0001). (B) Examples of larval homogenates derived from conventionalized fish following EHEC infection, plated on EHEC selective CHROMagar. Mauve, blue, and white colonies correspond to EHEC, other coliforms, and *Proteus* sp., respectively. (C) Colonization of conventionalized zebrafish with EHEC::mCherry following 2 (Ci), 8 (Cii), 16 (Ciii), and 24 h (Civ) of infection.

It has been reported that zebrafish acquire a microbiota which rapidly diversifies during early development (19, 37, 38). To test whether EHEC infection can be studied in the zebrafish model against the backdrop of the endogenous microbiota, we compared levels of EHEC in gnotobiotic fish, in which embryos are treated with mild bleach solution before hatching and being raised in sterile E3 medium, and in conventionalized fish, which consisted of bleached embryos transferred into a mixture of E3 medium and tank water following hatching in order to replenish the endogenous microbiota. Compared to the gnotobiotic fish results, initial colonization with EHEC was significantly decreased, but not entirely eliminated, in conventionalized fish (Fig. 8). Surprisingly, the colonization levels in conventionalized fish were very consistent, even though the compositions as well as levels of the colonizing microbiota differed considerably between individual animals (Fig. 8B). In the conventionalized fish, the bacterial burden expanded with increased incubation time and could easily be visualized from 16 hpi (Fig. 8C).

## DISCUSSION

Here, we establish zebrafish larvae as a new vertebrate model for EHEC infection. Maintenance of zebrafish is inexpensive, and their propagation and development are quick compared to other vertebrate animals, making them an attractive model organism for infection biology. Zebrafish have been used extensively to study bacterial infections, but few gastrointestinal infection models have been described to date, and our report provides, to our knowledge, the first extensive description of a foodborne infection model in zebrafish. Limitations of the zebrafish model are that the microbiota of humans and the microbiota of zebrafish are quite different, although they share some characteristics in that they act as a colonization barrier against pathogens and are stable and yet individually variable. *P. caudatum* is an ideal vector for foodborne infections: it is commonly used as a food source for young zebrafish, and interactions between E. coli and paramecia have been characterized previously (39). Another limitation of the current model is that the adaptive immune response is not yet fully mature at the larval stage (4 to 10 dpi), and so its contribution to infection remains to be elucidated. In agreement with earlier studies, we found no detrimental effect of EHEC-associated virulence factors on *P. caudatum* proliferation. E. coli is taken up into food vacuoles within seconds to minutes, depending on the density of suspended particles (40). Vacuoles gradually acidify from an initial pH of 8 to reach a pH of close to 1 (40), and EHEC have a half-life of approximately 150 min under those conditions. The bacterial passage through an acidifying compartment and the subsequent release into the zebrafish foregut upon ingestion of paramecia mimic passage through the human GI tract and acidification in the stomach.

Upon ingestion, paramecia are degraded in the foregut and released EHEC rapidly colonize the mid-intestine. Although bacteria were initially observed both in foregut and mid-intestinal tract, EHEC shows a distinct preference for colonizing the mid-intestine, a site which contains three of the four principal cell types found in mammalian intestinal epithelium plus specialized enterocytes analogous to the M cells found in adult mammalian intestine (41). The site of infection displays sharp boundaries, with temporary colonization of the foregut during early infection and no colonization of the posterior intestine (Fig. 2 and 4). In contrast, colonization of infant rabbit is not as localized, with similar bacterial burdens found in ileum, cecum, and colon even at later time points (5). Whether EHEC displays any altered ability or preference to colonize different intestinal epithelial cell types early in the infection process is still unknown; however, the similarity between mammalian and zebrafish intestinal epithelial cells and the genetic tractability and optical properties of zebrafish will hopefully provide a useful tool to further dissect these interactions.

Human EHEC infection is known to cause a strong proinflammatory response, and neutrophil infiltration of the lamina propria and transmigration through the intestinal epithelium into the gut lumen have been described in monkey, piglet, and rodent models (5, 42, 43). This is a response to increased IL-8 production by the intestinal mucosae, although it has been a point of contention whether Stx or Toll-like receptor 5 (TLR5) recognition of H7 flagellin was the major factor inducing IL-8 secretion. *In vitro* and *ex vivo* studies using flagellar mutants demonstrated that IL-8 secretion is driven by exposure of epithelial cells to flagellar antigen and, to some extent, by TLR4-driven responses to lipopolysaccharide (LPS) (44, 45). In our model, Stx-negative EHEC is still capable of mounting a strong neutrophilic inflammation, which supports these data. While neutrophil depletion has been shown to increase the bacterial burden of Citrobacter rodentium in mouse (46) and targeting leukocyte adhesion factor with antibody reduces disease symptoms in rabbits (3), no direct link between neutrophil recruitment and bacterial clearance has been established for EHEC. The zebrafish immune system displays many similarities to that of mammals, with counterparts for most human immune cell types (47). The zebrafish innate immune system starts to develop as early as 24 h postfertilization (hpf) with primitive macrophages followed by neutrophils at 32 to 48 hpf. The development of the adaptive immune system lags, taking another 4 weeks to fully develop (48). This feature provides an opportunity to exclusively observe the innate immune reaction to an EHEC infection in our experimental system. We observed a significant increase in neutrophil recruitment to the tissue surrounding the infection site as early as 2 hpi, with a peak at around 8 hpi. Coincidentally, this also seems to coincide with bacterial burden (Fig. 2D), which is decreased at 3 hpi and gradually diminishes thereafter. However, a low level of persistence was still observed at the experimental endpoint of 24 hpi. Although the evidence is circumstantial, the timing of these two events suggests that bacterial clearance may be mediated, at least in part, by neutrophils. Although the issue is beyond the scope of this study, the zebrafish EHEC infection model will allow further, higher-resolution analyses for the study of EHEC-neutrophil interaction dynamics.

EHEC infection in infant rabbits is self-limiting, with a peak in bacterial burden at approximately 7 dpi and a decline in inflammation and bacterial burden thereafter (5). We observed a similar, albeit accelerated pattern in zebrafish, with peak inflammation at approximately 8 hpi. Bacterial burden declines over time, and interestingly, there were two distinct colonization patterns: approximately half the animals cleared the infection, while the other half displayed low but persistent colonization until the experimental endpoint, without signs of clearance (Fig. 2C). Although current license requirements prevented us from doing so here, future work will focus on following infections for longer periods to establish if EHEC is eventually fully cleared from these animals.

Following attachment to the host epithelia, EHEC regulates the coordinated expression of a range of bacterial effectors through the activity of virulence factors encoded by LEE1 to LEE5. The LEE1-encoded master regulator of the LEE pathogenicity island, Ler, has previously been shown in *in vitro* models to be activated early in infection and is regulated in part through bacterial attachment and sensing of fluid shear (49). The activity of the five LEE operons is also temporal in nature, with *in vitro* models demonstrating that by 3 hpi, LEE1 activity is downregulated whereas transcription of LEE3-carried and LEE5-carried genes is upregulated (50). Through the use of EHEC strains expressing a LEE1::gfp reporter, we were able to utilize the zebrafish model to visualize LEE1 activation *in vivo* during the early stages of infection and to correlate its expression with the EHEC colonization pattern. Our results demonstrate the importance of tight control of EHEC virulence gene regulation for successful colonization *in vivo*. Deletion of *adhE*, which posttranscriptionally affects the regulation of virulence genes through Hfq, suppresses production of the LEE-encoded T3SS and decreases colonization in a rabbit model (31). Decreased colonization was also recapitulated in the zebrafish model, with the levels of both LEE1 induction and bacterial burden decreased in an *adhE* mutant compared to the TUV 93-0 wild-type strain (Fig. 5). Our data further demonstrate that temporal regulation of LEE activation is important for successful colonization; deletion of *tolA* causes constitutive activation of the Rcs phosphorelay and expression of LEE-carried virulence genes outside the host environment (30). Despite overexpression of the T3SS, *tolA* mutants display decreased attachment to intestinal epithelial cells and reduced virulence in Galleria mellonella infection models. Our model recapitulated these findings in that, despite higher levels of LEE1 expression, the bacterial burden was significantly reduced compared to the Sakai wild-type strain level (Fig. 6). Both *adhE* and *tolA* strains caused significantly reduced morbidity in the zebrafish model compared to wild-type strain results (Fig. 7). These findings demonstrate that the zebrafish system faithfully reflects the contribution of T3SS, a key virulence factor, and its regulation to bacterial colonization and persistence. Our data also underpin that induction of virulence factors has to be carefully timed and fine-tuned in response to the host environment, with deregulated induction of virulence genes being just as detrimental to a pathogen’s fitness as loss of virulence factors.

In summary, the zebrafish represents a powerful model for the study of EHEC infection. The optical clarity of zebrafish larvae and the availability of transgenic fish lines, coupled with the use of bacterial reporter strains, enable simultaneous visualization of bacterial physiology and of the molecular dynamics of host-pathogen interactions in real time within a living organism. This model will therefore provide opportunities to study the molecular aspects governing EHEC colonization and pathogenesis *in vivo* at the single-cell level, with refined temporal resolution and sampling size.

## MATERIALS AND METHODS

### Bacterial strains and growth conditions.

The bacterial wild-type strains used in this study were EHEC O157:H7 strain 813, a derivative of Escherichia coli O157:H7 Sakai (a gift from S. Busby), and Escherichia coli O157:H7 TUV 93-0 (a gift from A. Roe), both of which are Stx-negative strains. The Δ*tolA* mutant was a derivative of strain 813 and was generated by gene doctoring (51). Mutant Δ*adhE* (31, 52) was derived from TUV 93-0 (53) and was received as a gift from A. Roe (University of Glasgow). For monitoring localization *in vivo*, strains were transformed with plasmid pDP151-mCherry (ampicillin resistant [Amp^r^]). To visualize induction of the LEE1-encoded regulator Ler, strains were transformed with plasmid LEE1::gfp or U9:*gfp* (promoterless negative control), both tetracycline resistant (Tet^r^) (49). Strains were grown in LB broth supplemented with 100 µg/ml ampicillin or 34 µg/ml tetracycline, where appropriate, at 37°C with gentle shaking.

### Fish maintenance and breeding.

The zebrafish (Danio rerio) strains used in this study were AB wild-type fish and transgenic fish of the Tg(*mpo*::*egfp*)^i114^ line that produce green fluorescent protein (GFP) in neutrophils (24). Adult fish were kept in a recirculating tank system at the University of Birmingham Aquatic Facility or at the UTHealth Center for Laboratory Animal Medicine and Care under conditions of a 14-h/10-h light/dark cycle at pH 7.5 and 26°C. Zebrafish care and breeding and experiments were performed in accordance with the Animal Scientific Procedures Act 1986, under Home Office project license 40/3681 (University of Birmingham) and Animal Welfare Committee protocol AWC-16-0127. Eggs were obtained from natural spawning between adult fish, which were set up in groups of 7 (4 females and 3 males) in separate breeding tanks. After collection of eggs, larvae were kept in a diurnal incubator under conditions of a 14-h/10-h light-dark cycle with the temperature maintained at 28.5°C. Embryos were raised in petri dishes containing E3 medium (5 mM NaCl, 0.17 mM KCl, 0.33 mM CaCl_2_, 0.33 mM MgSO_4_) supplemented with 0.3 µg/ml of methylene blue. From 24 hpf, 0.003% 1-phenyl-2-thiourea (PTU) was added to prevent melanin synthesis. During infections, larvae were maintained at 32°C. All zebrafish care and husbandry procedures were performed as described previously (54).

### Preparation and maintenance of gnotobiotic and conventionalized fish.

Gnotobiotic embryos were produced by stepwise bleaching of eggs with a 0.0045% sodium hypochlorite solution in E3 medium at 12 hpf, as previously described (54). Since the sodium hypochlorite solution leads to a hardening of the chorion, embryos were freed manually by dechorination. After bleaching, eggs were transferred and maintained in sterile E3 medium. To enable colonization of embryos by a conventional gut microbiota, embryos were transferred to untreated tank water directly obtained from the aquatic system used for maintenance of adult zebrafish following dechorination.

### Maintenance of *Paramecium caudatum*.

Paramecia were cultured at 22°C in 5-cm-diameter petri dishes with E3 medium containing E. coli MG1655 as a food source. To maintain the culture, 0.5 ml of an existing paramecium culture was passaged into 9.5 ml of fresh E3 medium containing 10^8^ CFU/ml of E. coli MG1655.

### Uptake and clearance of Escherichia coli O157:H7 by *P. caudatum*.

**T**he uptake of fluorescent EHEC by paramecia into vacuoles was observed by live imaging and on fixed samples under a fluorescence microscope. The acidification of the food vacuoles and subsequent degradation/clearance of bacteria were followed by additional staining with lysotracker red. To determine EHEC viability within *P. caudatum*, samples were removed from *P. caudatum* cultures at the indicated time points, lysed with 1% Triton X-100–phosphate-buffered saline (PBS), and homogenized, and serial dilutions in PBS were plated on CHROMagar O157 plates for enumeration of CFU.

### Foodborne and waterborne fish infections.

For infection experiments, bacteria were harvested by centrifugation at 6,000 × *g* for 2 min and adjusted to an optical density at 600 nm (OD_600_) of 1.0 (approximate concentration, 1 × 10^9^ bacteria/ml). *P. caudatum* was quantified using a hemocytometer and added to the suspension to give a concentration of 10^5^ paramecia/ml, and the reaction mixture was incubated for 2 h at 32°C. Following preincubation, paramecia were washed and added to zebrafish larvae (4 dpf) housed in 6-well plates to give the indicated bacterial concentrations. For waterborne infections, the indicated EHEC concentrations were directly prepared in E3 medium and added to zebrafish larvae (2 ml/10 zebrafish larvae/6-well plate). Following infections, zebrafish larvae were either anesthetized by adding tricaine (final concentration, 160 µg/ml) to 20 ml E3 medium and 2 ml of a 0.1 M sodium-bicarbonate solution to buffer the medium or euthanized by adding 1.6 mg/ml tricaine to the buffered medium. For microscopy, larvae were transferred to a 1.5-ml microcentrifuge tube, washed in PBS, fixed by adding 1 ml of 4% paraformaldehyde solution–PBS, and stored at 4°C in the dark until use. For mortality, larvae were infected using a modified waterborne infection route in which larvae were maintained in a solution of E3 medium containing the indicated concentration of EHEC for the duration of the study.

### Imaging of infected fish.

For live imaging, infected anesthetized larvae were positioned in 96-well glass-bottom plates and covered and immobilized with 1% low-melting-point agarose solution. A 200-µl volume of E3 medium containing 160 µg/ml tricaine was added to cover the immobilized larvae. Live imaging was performed at 32°C and 80% humidity. A Zeiss Axio Observer inverse microscope equipped with a 10× objective was used for acquisition of 2 fluorescent channels and 1 differential interference contrast (DIC) channel. The four-dimensional (4-D) images produced by the time-lapse acquisitions were processed, clipped, examined, and interpreted using Zen 2 software (Zeiss). Maximum intensity projection was used to project developed Z-stacks, and files were exported in tiff format for images or in mov format for QuickTime movies. Final figures were assembled with CorelDrawX5.

### Determination of bacterial burden in infected fish.

After euthanasia, larvae were transferred to individual microcentrifuge tubes and disintegrated by repeated pipetting and vortex mixing in 200 µl PBS supplemented with 1% Triton X-100. Serial dilutions of bacterial suspension were plated onto selective CHROMagar O157 plates, and plates were incubated at 37°C for 16 h. Colonies identified as EHEC (mauve appearance on selection plate) were quantified for analysis.
