# Supplementary figures and images for: Zebrafish (Danio rerio) as a Vertebrate Model Host To Study Colonization, Pathogenesis, and Transmission of Foodborne Escherichia coli O157
Source: mSphere. 2017 Sep 20;2(5):e00365-17. doi: 10.1128/mSphereDirect.00365-17 (PMC5607324; doi:10.1128/mSphereDirect.00365-17)

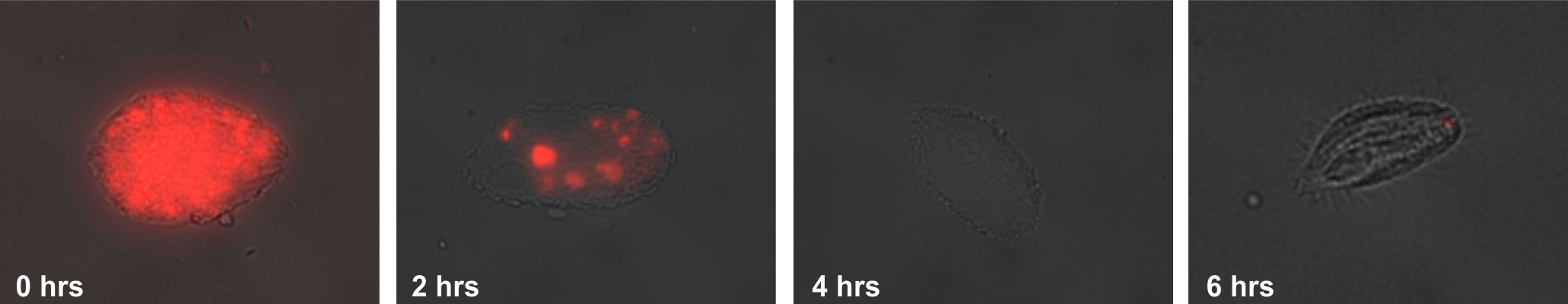

Supplement: FIG S1 [file sph005172365sf1.tif]

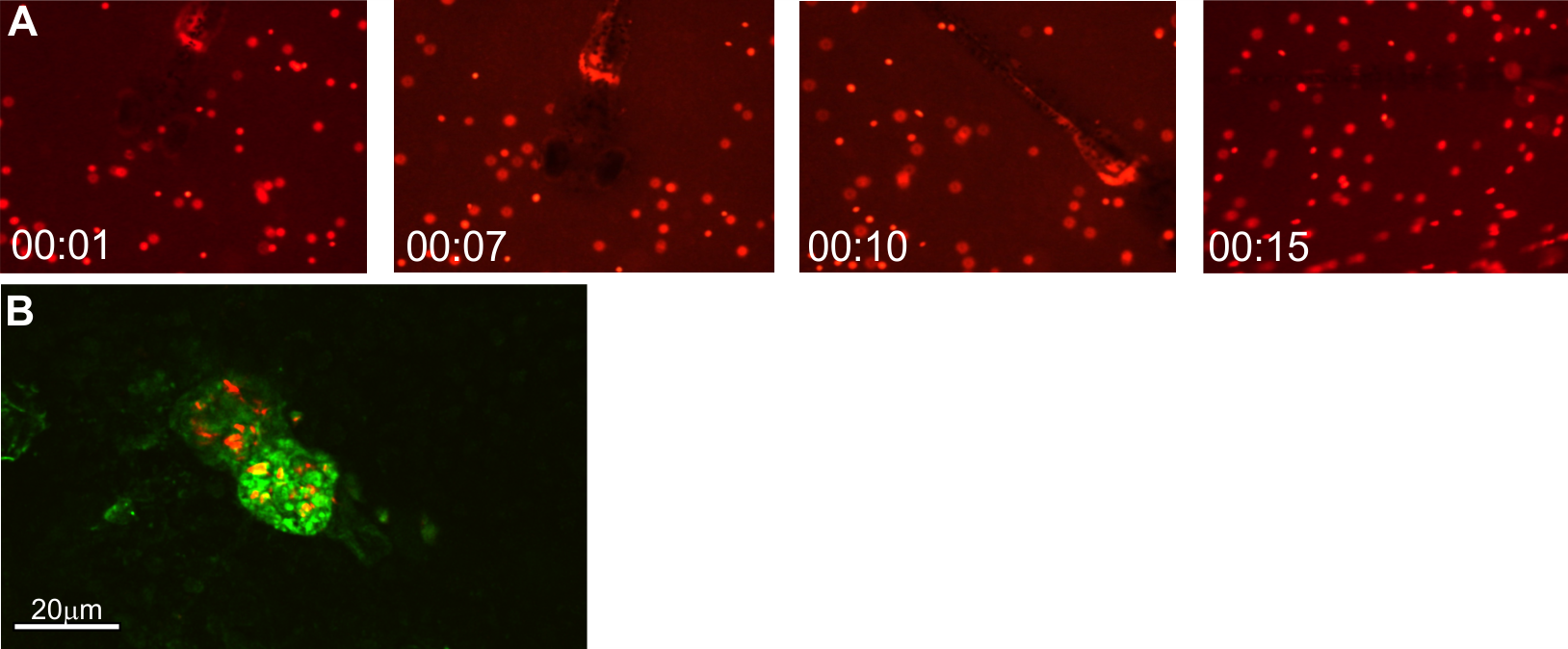

Supplement: FIG S2 [file sph005172365sf2.tif]
